# The value of the World Conferences on Research Integrity: perspectives from Peru

**DOI:** 10.1080/20961790.2021.1972906

**Published:** 2021-10-13

**Authors:** Mariela Dejo-Vásquez, Roxana Lescano, Juan Guillermo Pérez-Carreño

**Affiliations:** a Universidad de Lima, Lima, Peru; b Asociación Peruana para el empleo y bienestar de animales en investigación y docencia (ASOPEBAID), Lima, Peru; c Universidad del Rosario, Bogotá, Colombia

“The World Conferences on Research Integrity (WCRIs), six to date, were initiated at a time when researchers in various countries were at quite different stages in their thinking about research integrity. Over the course of the past decade, the global conferences have reflected and documented signifi­cant changes in the way research integrity is viewed and supported” [1].

Attracting researchers, university officials charged with handling misconduct, government representatives, and research funders, the WCRIs “foster the exchange of information and discussion about responsible conduct of research” [2]. Over the course of the first five WCRIs, attendance grew from 275 participants from 47 countries in 2007 to 836 participants from 52 countries in 2017 [3]. The 6th WCRI gathered a record number of 701 attendees from over 50 countries in all regions of the world and from various disciplines, and roles, including researchers, teachers, leaders of funding agencies, government officials, journal editors, senior admini­strators and research students [4]. The next WCRI is planned for Cape Town, South Africa in 2022.

Each WCRI has been the cradle of collaborative networks in the field of research integrity. For example, the European Network of Research Integrity Offices (ENRIO) was formed in 2008 after the first WCRI in Lisbon, with the aim of promoting the exchange of information and experiences. The first ENRIO meeting had eight attendees. As of 2021, ENRIO has 31 member organizations from 23 European countries. This network shares information across its members and has developed resources such as a manual for investigating misconduct in research [5]. Similarly, the Asia Pacific Research Integrity (APRI) and African Research Integrity (ARIN) networks emerged and were enhanced through connections made *via* the WCRI (Courtesy of Zoë Hammatt—president of Z Consulting, LLC. Former Director of the Division of Education & Integrity, US Office of Research Integrity, and Francis Kombe—University of KwaZulu-Natal-SA, Kenia and ARIN Steering Committee Member). Members of the African network submitted a successful bid to host the 7th WCRI in Cape Town, South Africa. Due to the COVID-19 pandemic, a digital bridging event occurred in 2021 [6] and an in-person event in Cape Town is planned for 2022 [7].

In large part due to the forum created by the WCRIs, professionals from various disciplines and organizations have come together to establish common standards and definitions related to research integrity [8]. Networks and other groups have emphasized diverse topics such as research misconduct, authorship, data management, collaborative science, peer review, conflicts of interest, and others [9]. These topics form part of evolving discussions and research in the field of research integrity. Such integrity is essential to ensuring responsible science and practice in all disciplines, reinforcing public confidence in research of all kinds. As the quality of higher education in a country becomes increasingly important for a country’s development, the trustworthiness of research takes on heightened importance.

The Inter-American Network, formed at the 6th WCRI held in Hong Kong, China with members from Brazil, Colombia, Peru, Chile, and the US has contributed to raising awareness of research integrity issues in Latin America. The network established standards for the responsible conduct of research through the joint project “Generation of Recommendations in Scientific Integrity” and has undertaken the translation to Spanish of the Hong Kong Principles, among other initiatives.

The participation of Peruvians in the WCRIs has catalyzed the sharing of knowledge in Peru. Such sharing has been reflected in meetings and events to discuss research integrity, formation of networks, and published articles by Peruvians on plagiarism, predatory journals, good practices, and other topics. Unfortunately, only a few representatives from Latin America have participated thus far in WCRIs, networks, and local events. Thus, we believe more space should be created, particularly in Peru and other countries where systems for research integrity are still being established. Such space should offer a safe place to share experiences, initiatives, and concerns about research and academic integrity. Perhaps most important is coming together to conduct training, reinforce mentoring opportunities, and enhance mechanisms for investigating misconduct and promoting integrity in Peru and across Latin America.

The culture of research is on a steady rise in Peru. Efforts to ensure the quality of Peruvian education, such as Law 30220, approved in 2014 [10], established conditions to regulate the quality of higher education and research. One of the requirements of this law is that each research university must have certain elements in place. These include a code of ethi­cs, software for detecting similarities and plagiarism, and processes for evaluating plagiarism in student work. In addition, the regulations of the National Council for Science and Technology of Peru (National Council) and its Declaration of Principles for Scientific Integrity form the foundation for strengthening Peruvian science and technology [11]. As of August 2021, there are four universities with dedicated offices for research integrity, policies and training, out of 139 universities in Peru. It is not surprising that they emerge in rankings as the best universities in the country with the highest production in research. Likewise, various Peruvian academic institutions have participated in the Asia Pacific Integrity Principles project, an initiative of the Royal Melbourne Institute of Technology, led by Dr. Daniel Barr and the late Dr. Paul Taylor. This innovative pro­ject was designed to reach consensus on principles of research integrity across the various Asia-Pacific Economic Cooperation (APEC) economies as a means of facilitating more meaningful exchange between researchers in different countries. A publication is in process.

Research-intensive universities must continue to expand awareness and join efforts to enhance integrity in all disciplines. In Peru, the needs of resear­chers have sparked interest among scientists and academic institutions, leading to heightened awareness in this area.

Two specific efforts in Peru are worthy of mention. First, since 2017, the National Council has required researchers to obtain a certificate of competence in responsible conduct of research. This certificate has been a requirement to register in the National Registry of Science and Technology and apply for competitive funds. In 2019, the National Council also implemented a scientific integrity code. Another initiative led by the National Council seeks to strengthen local scientific journals, supporting their process to achieve indexing in Scopus®, Elsevier’s curated abstract and citation database. Secondly, the newly created National Superintendency of Higher Education, which aims to raise the standards of Peruvian university education, has ensured that licensed universities have a code of ethics and meet the requirement of similarity detection software and processes for assessing plagiarism.

While these and other efforts are critical to Peru’s evolution in terms of fostering a culture of integrity and innovation, we still have a long way to go. Perhaps one of our next steps is to embed such requirements into practice.

From our experience in the Peruvian context, some suggestions emerge on how to overcome barriers and find creative solutions to nurturing an environment of integrity in developing countries, such as: building consensus among research partners, getting institutional leaders and faculty on board, especially for strong mentoring, using what resources you have, and focusing on training through webinars, among others.

Other Latin American countries have carried out a parallel process of developing guidelines. For example, the Policy on Ethics, Bioethics and Scientific Integrity was approved in Colombia in 2017. The policy’s purpose was to propose a roadmap for the different institutions that form part of the National System of Science, Technology and Innovation to consolidate a scientific culture based on ethics, bioethics and scientific integrity [12].

We believe that integrity emerges from principles that are grounded in the training and moral conscience of the researcher. Integrity is inextricably linked to personal convictions and community, societal and national beliefs, as well as researchers’ relationship to each of these and the environment. Researchers cannot dissociate themselves from the social processes and context of their projects [13]. It would seem to be the responsibility of institutions, funders, government leaders and legislators to incorporate and embrace science as a factor of developmental progress, particularly in developing countries where this represents an important measure of development.

We also believe that the WCRIs serve as an exemplary vehicle for uplifting the spirit of integrity in research around the world. In addition to offering a forum for discussion of these and other issues, these conferences have led to the development of guidelines for responsible research, including the Singapore Statement [14]. This concise statement has helped set the tone for the first training course on Responsible Conduct of Research in Peru and for the drafting of academic codes and the integrity code of the National Council for Science and Technology. Along with other tenets produced by the WRCIs, such as the Montreal Statement and Hong Kong Principles [15], we aspire to bring these and other important international guidelines to the attention of research teams in Peru and elsewhere in Latin America.

Mariela Dejo-Vásquez*Universidad de Lima, Lima, Peru*mcdejo@ulima.edu.peRoxana Lescano*Asociación Peruana para el empleo y bienestar de animales en investigación y docencia (ASOPEBAID), Lima, Peru*Juan Guillermo Pérez-Carreño*Universidad del Rosario, Bogotá, Colombia*Juan Guillermo Pérez-Carreño was attached to Universidad del Rosario during preparation of this manuscript. As of the date of submission, he is working for Janssen, a pharmaceutical company of Johnson & Johnson.
